# Comparing algorithms for assessing upper limb use with inertial measurement units

**DOI:** 10.3389/fphys.2022.1023589

**Published:** 2022-12-19

**Authors:** Tanya Subash, Ann David, StephenSukumaran ReetaJanetSurekha, Sankaralingam Gayathri, Selvaraj Samuelkamaleshkumar, Henry Prakash Magimairaj, Nebojsa Malesevic, Christian Antfolk, Varadhan SKM, Alejandro Melendez-Calderon, Sivakumar Balasubramanian

**Affiliations:** ^1^ Department of Bioengineering, Christian Medical College, Vellore, India; ^2^ Department of Mechanical Engineering, Indian Institute of Technology Madras, Chennai, India; ^3^ Department of Applied Mechanics, Indian Institute of Technology Madras, Chennai, India; ^4^ Department of Physical Medicine and Rehabilitation, Christian Medical College, Vellore, India; ^5^ Department of Biomedical Engineering, Lund University, Lund, Sweden; ^6^ School of Information Technology and Electrical Engineering, University of Queensland, Brisbane, Australia; ^7^ School of Health and Rehabilitation Sciences, The University of Queensland, Brisbane, Australia; ^8^ Jamieson Trauma Institute, Metro North Hospital and Health Service, Brisbane, Australia

**Keywords:** hemiparesis, machine learning, sensorimotor assessment, upper-limb rehabilitation, upper-limb use, wearable sensors

## Abstract

The various existing measures to quantify upper limb use from wrist-worn inertial measurement units can be grouped into three categories: 1) Thresholded activity counting, 2) Gross movement score and 3) machine learning. However, there is currently no direct comparison of all these measures on a single dataset. While machine learning is a promising approach to detecting upper limb use, there is currently no knowledge of the information used by machine learning measures and the data-related factors that influence their performance. The current study conducted a direct comparison of the 1) thresholded activity counting measures, 2) gross movement score,3) a hybrid activity counting and gross movement score measure (introduced in this study), and 4) machine learning measures for detecting upper-limb use, using previously collected data. Two additional analyses were also performed to understand the nature of the information used by machine learning measures and the influence of data on the performance of machine learning measures. The intra-subject random forest machine learning measure detected upper limb use more accurately than all other measures, confirming previous observations in the literature. Among the non-machine learning (or traditional) algorithms, the hybrid activity counting and gross movement score measure performed better than the other measures. Further analysis of the random forest measure revealed that this measure used information about the forearm’s orientation and amount of movement to detect upper limb use. The performance of machine learning measures was influenced by the types of movements and the proportion of functional data in the training/testing datasets. The study outcomes show that machine learning measures perform better than traditional measures and shed some light on how these methods detect upper-limb use. However, in the absence of annotated data for training machine learning measures, the hybrid activity counting and gross movement score measure presents a reasonable alternative. We believe this paper presents a step towards understanding and optimizing measures for upper limb use assessment using wearable sensors.

## 1 Introduction

The accurate evaluation of the real-world impact of a neurorehabilitation intervention is crucial to gauge its true value. Thus, there is a growing interest in quantifying how much and how well patients use their affected upper limb(s) outside of therapy; here, ‘how much’ refers to the amount of use of the upper-limb, and ‘how well’ refers to the movement quality during use. The shortcomings of current questionnaire-based assessments of upper limb use in daily life (Shephard, 2003) have led to a surge in the use of wearable sensors for this purpose. Several research groups have explored different sensing modalities (Bailey et al., 2014; Friedman et al., 2014; Laput et al., 2016; Malešević et al., 2019; Lum et al., 2020; David et al., 2021a; De Lucena et al., 2021; Tsai et al., 2021) and data analysis techniques (Bailey et al., 2014), (Lum et al., 2020), (Uswatte et al., 2000; De Lucena et al., 2017; Leuenberger et al., 2017; David et al., 2021b) for assessing the amount and quality of upper limb use outside the clinic. The work in the current paper focuses on ‘how much’ an upper limb is used.

Among the various constructs associated with upper limb functioning in daily life, the most fundamental one is the *upper limb use*—a binary construct indicating the presence or absence of a voluntary, meaningful movement or posture (David et al., 2021b); it is essential for deriving the other constructs in upper limb functioning (David et al., 2021b). Upper limb use assessment focuses only on measuring willed movements or postures of functional significance. Identifying such movements is a relatively trivial task for a human observing a subject performing various movements. A human’s ability to relate to the movements being observed allows him/her to make judgements about the nature of a subject’s movements. However, doing this in an autonomous manner using technology is challenging.

The information gathered from measurement systems during everyday life from community-dwelling patients is limited due to constraints on the sensors’ size, wearability, ergonomics, and cosmetics. The most popular sensing modality is inertial sensing using inertial measurement units (IMUs) in the form of wristbands (Bailey et al., 2014), (David et al., 2021a), (Laput and Harrison, 2019), which measure linear acceleration and angular velocities of the forearm. Various measures have been developed to quantify upper limb use from only wrist-worn IMU data (Bailey et al., 2014), (Lum et al., 2020), (Uswatte et al., 2000), (Leuenberger et al., 2017), (De Lucena et al., 2017). Measures to quantify upper limb use using additional inertial measurements from other body segments exist in the literature (Uswatte et al., 2000), (Regterschot et al., 2021) but this study restricts its attention to only wrist-worn inertial measurements due to its popularity and practicality. The measures for detecting upper limb use employing only wrist-worn IMU data can be grouped into three types: 1) Thresholded activity counting (Bailey et al., 2014), (Uswatte et al., 2000), (De Lucena et al., 2017), 2) Gross movement score (Leuenberger et al., 2017), and 3) machine learning (Lum et al., 2020). Currently, thresholded activity counting-based measures are the most popular approach for quantifying upper-limb use (Bailey et al., 2014), (Uswatte et al., 2000), (De Lucena et al., 2017). These measures use quantized linear acceleration (often gravity-subtracted) to compute ‘activity counts’, which is then thresholded to quantify the presence or absence of a functional movement at any given time instant. The gross movement (GM) measure proposed by Leuenberger et al. (2017) uses estimates of forearm orientation to decide on the functional nature of upper limb movements. Bochniewicz et al. (2017) and Lum et al. (2020) used accelerometer data and tested different machine learning methods as a measure of upper limb use.

A comparison of the performance of these different upper limb use measures has also recently appeared in the literature (Lum et al., 2020), (Subash et al., 2022). Lum et al. compared the performance of thresholded activity counting to the different machine learning measures (Lum et al., 2020) on data collected from 10 unimpaired and 10 stroke survivors. They found that the random forest classification algorithm had an overall accuracy of greater than 90%, compared to about 72% for the activity counting methods. They observed that activity counting overestimated upper limb use by indiscriminately picking up both functional and non-functional movements. In our recent study, we made a similar observation comparing the activity counting with the GM measure (Subash et al., 2022) using data from 10 unimpaired subjects (David et al., 2021a). Activity counting had good sensitivity but poor specificity, compared to GM, which had a lower sensitivity but good specificity for functional movements. One approach to take advantage of the strengths of the activity counting and GM algorithm is to combine them into a hybrid algorithm. One such algorithm, termed GMAC (GM + activity counting), is introduced in the current study. A direct comparison of all these different measures (existing ones like the thresholded counts, GM, machine learning, and new ones like GMAC) of upper limb use is currently missing in the literature. Such a comparison on the same dataset can help delineate the pros and cons of these different measures. This comparison will also help to verify the claims made by Lum et al. (2020), and thus, evaluate the generalizability of their results.

Machine learning methods are data-driven approaches. The nature of the training data will impact the performance and generalizability of their result. However, this influence of training data on upper limb use detection has not been investigated in the current literature. Factors such as the relative amounts of functional *versus* non-functional movements and the types of tasks in a dataset can significantly impact a model’s training and testing accuracy. Furthermore, there has also been little work on the interpretability of machine learning methods used for upper limb use assessment. The current methods are black boxes that use a set of handcrafted features based on the authors’ intuition and experience about the nature of the movements of interest. Understanding the relative importance of the features could improve the interpretability of these methods. Comparing classifiers with a reduced number of features can indicate which features are more important than the others. Analyzing the relationship between important features and interpretable features (such as arm orientation) can lend insight into the basis for the classification.

This study aims to answer the following research questions to push forward the *status quo* in upper limb use assessment.

Q1. What is the best measure or algorithm for measuring upper-limb use, among the existing and the new measures introduced in this study?

Q2. What information about a subject’s movement do machine learning algorithms rely on to measure upper-limb use?

Q3. Given that machine learning algorithms need to be trained and validated *a priori* to measure upper-limb use, how does the nature of the training and validation datasets affect a machine learning algorithm’s performance?

The additional focus on machine learning measures (Q2 and Q3) is due to the increasing interest in these measures, and the current lack of understanding of these measures in this application.

This study uses an annotated dataset collected from our previous study (David et al., 2021a), which consists of data from 10 unimpaired and five stroke survivors performing a set of activities involving arm and hand movements.

## 2 Methods

### 2.1 Data collection

#### 2.1.1 Device

**FIGURE 1:**
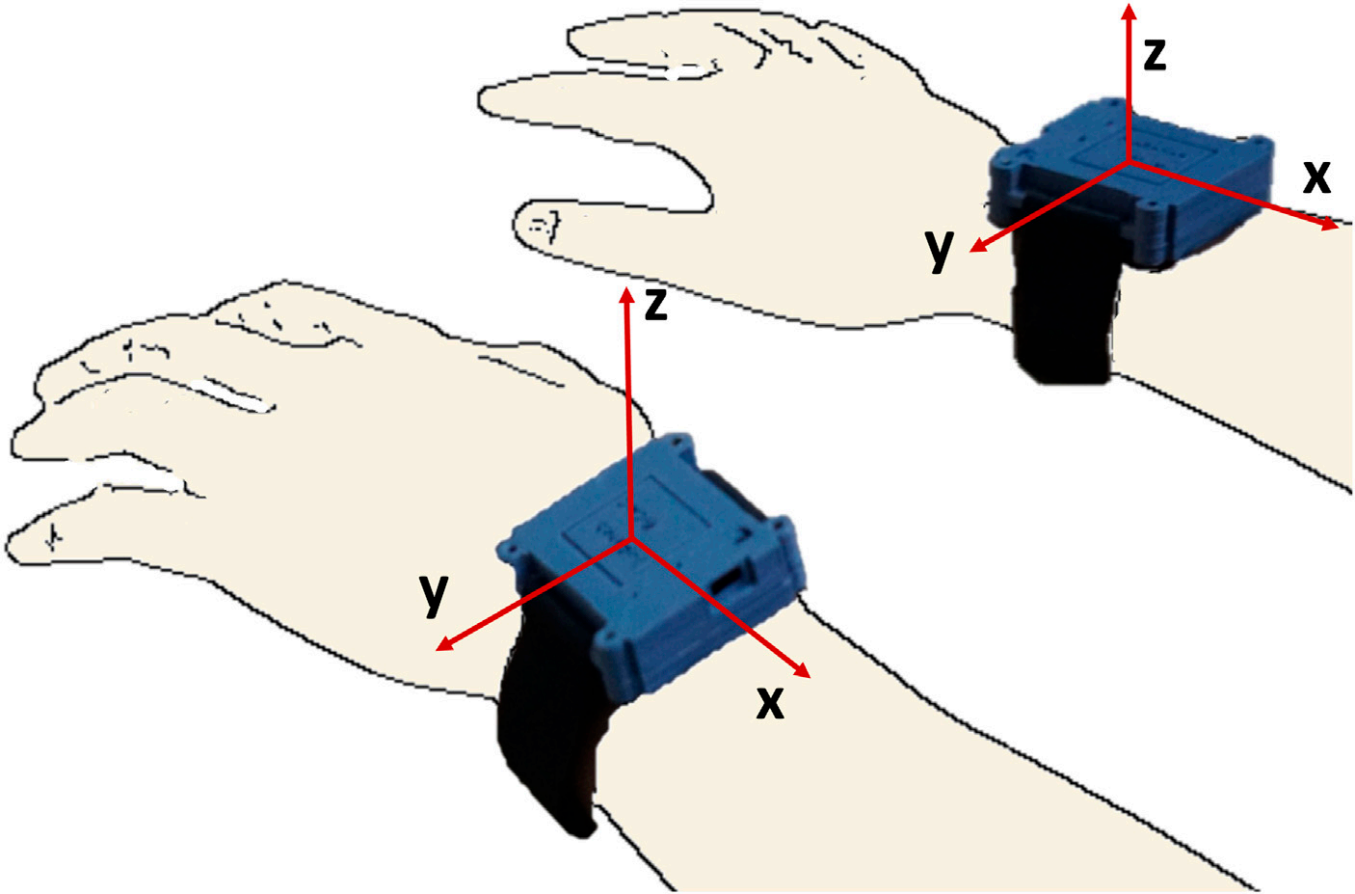
IMU watches with the axes of the accelerometers for each of the two watches.

Data from a previous study (David et al., 2021a) (approved by the institutional review board of Christian Medical College (CMC) Vellore, IRB Min. No. 12321 dated 30.10.2019; CTRI/2018/09/015,648) on the in-clinic validation of a wrist-worn sensor band, dubbed ‘IMU-Watch’, was used for this analysis. The IMU-Watches log triaxial accelerometer, gyroscope, and magnetometer data at 50 Hz in synchrony. The directions of the axes of the accelerometers in the IMU Watches are shown in Figure 1.

#### 2.1.2 Participants

As part of the study (David et al., 2021a), 10 unimpaired individuals and five stroke survivors with hemiparesis were recruited. The inclusion criteria for the patients with hemiparesis were: 1) no severe cognitive deficits (Mini-Mental State Examination score (MMSE) higher than 25); 2) Manual Muscle Test (MMT) grade higher than two of the upper-limb muscles (shoulder abductors, elbow flexors, elbow extensors, wrist extensors, finger flexors, hand intrinsics) (Naqvi and Sherman, 2022); 3) age between 25–70°years; can actively achieve 4) at least 30 elevation of the arm against gravity in the shoulder joint with the elbow extended, 5) 20 wrist extension against gravity, and 6) 10 finger extension (proximal metacarpophalangeal and interphalangeal) of at least one finger against gravity; 7) ability to open the hand in any position to accommodate a small ball (diameter of 1.8 cm) in the palm; and 8) willingness to give informed consent. Patients were recruited through the inpatient Occupational Therapy unit of CMC Vellore.

The inclusion criteria for unimpaired controls were: 1) no prior history of upper limb movement problems due to neurological conditions; 2) no current difficulty in upper-limb movements; 3) age between 25 and 70 years; and 4) willingness to give informed consent. Subjects who had pain while moving the upper limb and/or allergy to the plastic material used for the IMU-watch casing and straps were excluded from the study.

#### 2.1.3 Tasks

Participants performed various functional tasks (listed in Table 1) while wearing an IMU-Watch on each arm. The recordings for the tabletop and non-tabletop tasks were taken in two separate sessions, each lasting no more than 15 min. Control subjects performed all the tasks, but stroke survivors only completed a subset of these tasks because of difficulty in performing some tasks. Subjects mimicked movements 3–4 times for tasks such as eating or drinking.

**TABLE 1:** Tasks performed while wearing the IMU watches.

Tabletop tasks	
Write using a pen	Type on a keyboard
Make a call using a mobile phone	Button a shirt
Drink from a glass	Drink from a teacup with handles
Open a bottle	Comb your hair
Wipe a table	Fold a towel
Eat from a plate using your hands	Eat from a bowl using a spoon
Non-Tabletop Tasks	
Walk 25 m	Open a door
Hit a light switch	

#### 2.1.5 Ground truth labelling

The entire experiment was videotaped using a webcam connected to a PC time-synchronized with the IMU-Watches. Two therapists annotated the videos twice with 1 week’s gap between each annotation, using a custom software that allowed them to mark selected periods in the videos with pre-specified labels. The annotators were instructed to comply with FAABOS (Uswatte and Qadri, 2009) a coding scheme designed to quantify amount of arm activity from video recordings. It has been used in studies to validate sensor-based methods of classification of functional and non-functional use (Lum et al., 2020), (Bochniewicz et al., 2017), (McLeod et al., 2016) or between different activities (Totty and Wade, 2018). FAABOS provides definitions and examples to classify movements into four classes, based on the functional nature and task-relatedness of the movements, namely, task-related functional, non-task-related functional, non-functional, and no activity or movement. For this analysis, the annotators were asked to reduce the four classes to a binary classification indicating functional (e.g., reaching, eating, adjusting glasses) and non-functional movements (e.g., arm swing during walking, tic, tremor, passive movements, rests). In addition to the FAABOS annotations, the data was also marked to identify periods of predominantly ‘Hand’ movements (e.g., writing, typing), ‘Arm’ movements (includes tasks that involved use of both arms and hands, e.g., wiping, drinking), and ‘Non-Functional’ movements to reflect the type of functional movements carried out by the participants during the experiment. The epochs corresponding to the different tasks were also annotated in the data.

#### 2.1.6 Dataset preparation

The final dataset used for the analysis was prepared in the form of a table with the columns and rows corresponding to different features and time stamps, respectively. The different columns of this dataset include the following for each arm.

*Re-sampled sensor data:* Triaxial accelerometer, gyroscope, and magnetometer was recorded approximately at 50 Hz. The raw data from the watches were re-sampled to 50 Hz using zero-order hold interpolation to account for any missing data.

*Yaw and pitch angles of the IMUs:* Yaw and pitch angles of the forearm are estimated from the raw 50 Hz data with respect to an earth-fixed reference frame using the Madgwick algorithm (Madgwick et al., 2011). Offset correction was done on the raw gyroscope data before using the Madgwick algorithm by first identifying “rest” and “move” periods. A rest period is at least 10s long where the signal variance is less than 0.15 deg/s on each gyroscope axis. The mean angular velocity in each axis during a rest period was computed as a gyroscope offset value. This offset value is subtracted from the raw gyroscope data, starting from the current rest period until the next rest period to reduce gyroscopic drift. A fifth-order median filter was applied to the accelerometer data to remove sharp jumps and outliers.

*Annotations:* Four columns that correspond to functional use annotation marked twice by the two annotators, were included. Two other columns indicate the tasks and arm/hand use. All annotations were saved at the video frequency of 30 Hz. The annotations were included in the dataset after up-sampling them to 50 Hz using zero-order hold interpolation.

Each row in the dataset is time-stamped. The dataset also includes a column with subject identifiers to differentiate data between different subjects.

### 2.2 Comparison of Measures

**FIGURE 2:**
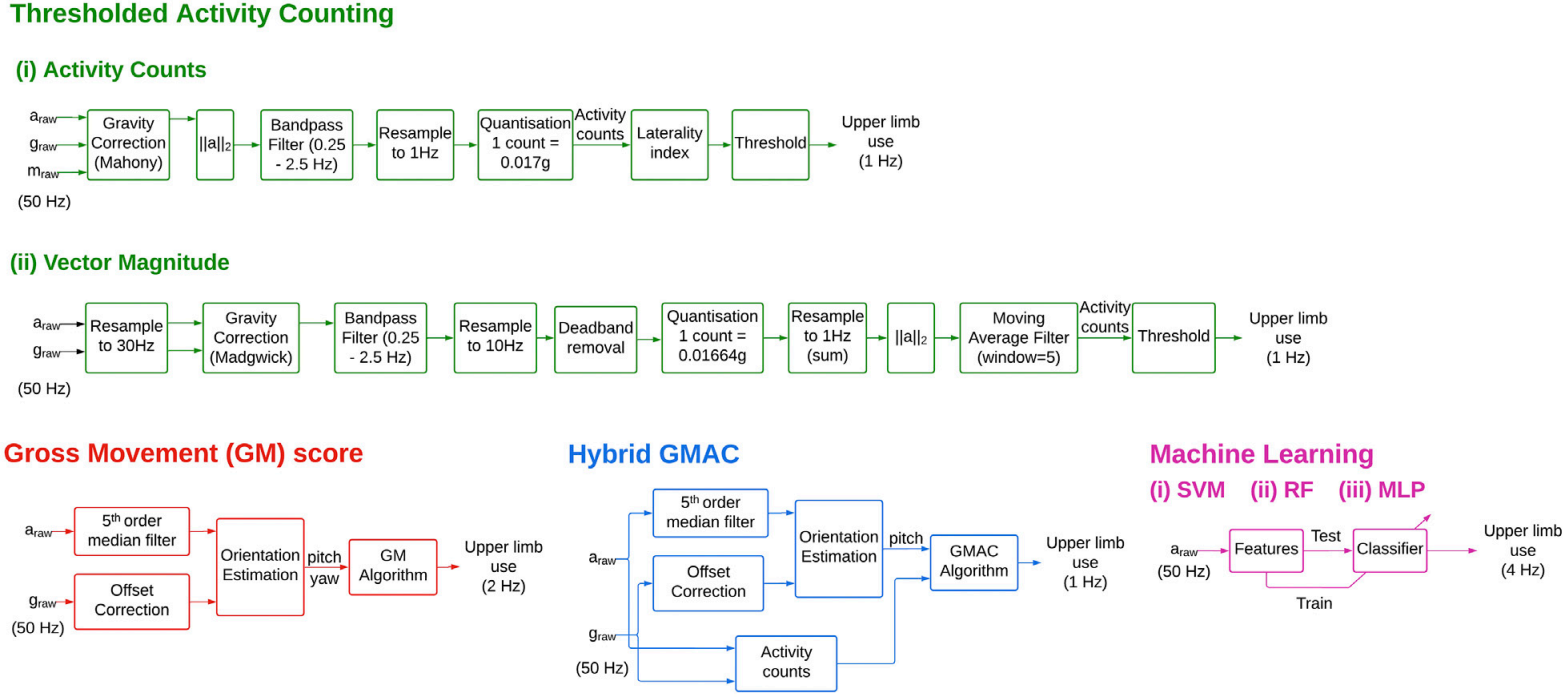
Block diagrams for the different UL use measures. These different blocks depict the essential steps involved algorithm. The details of the implementation of these steps can be found in the shared code repository.

The current study compared different measures (shown in Table 2) reported in the literature, along with two new measures (GMAC and MLP, described later in this section). The data processing pipeline (Figure 2) for these algorithms was implemented with an interest to remain true to the original work. Where the source material lacked sufficient detail, the processing steps and their associated parameters were chosen empirically as detailed below. The outputs of the different measures were compared with the manual annotations which served as ground truth.

**TABLE 2:** Upper limb use measures that were compared.

	Measure	Proposed by
1	Thresholded Activity Counting	
	Vector Magnitude (VM)	Bailey et al., (2014)
	Activity Counts (AC)	De Lucena et al. (2017)
2	Gross Movement (GM)	Leuenberger et al. (2017)
3	Hybrid GM and TAC (GMAC)	This study
4	Machine Learning	
	Random Forests (RF)	Lum et al. (2020)
	Support Vector Machine (SVM)	Lum et al. (2020)
	Multi-Layer Perceptron (MLP)	This study

#### 2.2.1 Thresholded Activity Counting (TAC)

The amount of acceleration is thresholded using a measure-specific threshold to estimate upper limb use. The computational simplicity of this measure makes it a quick and popular approach (Bailey et al., 2014), (Uswatte et al., 2000), (De Lucena et al., 2017). However, while an increased amount of acceleration most likely correlates with increased upper-limb use, the feature is not unique to functional movements, and thus overestimates upper limb use. Two types of TAC measures were evaluated in this study: activity counting (De Lucena et al., 2017), and vector magnitude (Bailey et al., 2014).

##### 2.2.2 Activity Counts

This measure was proposed by de Lucena et al. (De Lucena et al., 2017). The effects of gravity are removed from the accelerometer data using the 9DOF IMU data with the Mahony algorithm (Mahony et al., 2008). The magnitude of the gravity-subtracted acceleration data is then bandpass filtered between 0.25 Hz and 2.5 Hz using a fourth order Butterworth filter. The magnitudes are down-sampled to 1 Hz by taking its mean using non-overlapping 1 s bins. The counts are computed by quantizing the magnitudes by 0.017 g. A laterality index is calculated using the counts from both arms and thresholded to produce binary signals that indicate use and non-use of both arms. Laterality greater than −0.95 denotes dominant or non-affected arm use, and an index lesser than 0.95 denotes non-dominant or affected arm use.

##### 2.2.1.2 Vector Magnitude

This measure proposed by Bailey et al. generates counts at 30 Hz using the Actigraph Activity Monitor (Bailey et al., 2014). In the current study, the counts were generated from raw acceleration data re-sampled to 30 Hz to match the Actigraph sampling rate. This enabled the direct application of the methods presented by BrØnd et al. (2017). The proprietary Actigraph filter was substituted for the Madgwick filter used with 6DOF IMU data. The gravity-corrected acceleration data were bandpass filtered between 0.25 Hz and 2.5 Hz using a fourth order Butterworth filter and re-sampled to 10 Hz. The data was then dead-band filtered using the thresholds ±0.068 g and summed for every 1s bin. The 2-norm of the acceleration vectors was then computed. A moving average filter with a window size of 5s with a 4s overlap was applied, resulting in the counts at 1 Hz. The counts were filtered using a zero threshold to obtain the binary signal corresponding to upper-limb use.

#### 2.2.2 Gross Movement (GM) measure

GM measure (Leuenberger et al., 2017) is computed for moving windows of 2s with a 75% overlap, resulting in upper limb use estimates at 2 Hz. The yaw and pitch angles were computed using the Madgwick algorithm from the raw acceleration and gyroscope data. If in a 2s window, the overall absolute change in yaw and pitch angles is higher than 30° and the absolute pitch of the forearm is within ±30° , GM is defined as 1 (indicating functional use), else it is 0. The GM measure exploits the nature of most functional movements to occur in this ‘functional space’, i.e., in the region in front of subject around his/her chest height.

$$GM\left(n\right)=\left\{\begin{array}{cc} 1, & if\;\left(\Delta yaw+\Delta pitch\right)>30{}^{\circ}\;\;and\;\left|pitch\right|<30{}^{\circ}\\ 0, & otherwise \end{array}\right.$$

#### 2.2.3 Hybrid GM and TAC (GMAC)

The TAC measures are known to be highly sensitive while having very low specificity, and GM is highly specific but not sensitive (Subash et al., 2022). To address their individual shortcomings, we propose a hybrid measure—GMAC—that combines the essential elements of TAC and GM measures. It employs counts using the vector magnitude measure with a modified GM measure; the counts were used instead of the absolute change in yaw and pitch angles. Counts were generated at 1 Hz, and the mean pitch is computed for every 1s bin. Upper limb use is defined as one when mean pitch is within ±30° and counts is more than 0, else it is 0.

$$GMAC\left(n\right)=\left\{\begin{array}{cc} 1, & if\;AC>0\;\;and\;\left|pitch\right|<30{}^{\circ}\\ 0, & otherwise \end{array}\right.$$

#### 2.2.4 Machine Learning

The TAC and the GM measures are designed to exploit the differences in functional and non-functional movements as measured by an IMU and intuited by a human observer. In contrast, supervised machine learning methods are data-driven and use an annotated dataset to learn the statistical differences between functional and non-functional movements. There is currently limited work using machine learning methods for detecting upper-limb use. presented a comparative analysis of supervised machine learning methods and settled on a random forest method Lum et al. (2020).

The machine learning methods train models using the ground truth data, i.e., the ‘functional use’ annotations by the human therapists. At each time instant, a single ground truth label was derived as the majority of the four ground truth labels corresponding to two markings by two annotators. In the case of a tie, the instant was labelled as non-functional. We trained and tested two types of models, inter-subject and intra-subject models, with different machine learning methods.

#### 2.2.4.1 Features

Eleven features proposed by Lum et al. (2020) were computed from the 50 Hz triaxial accelerometer data (Lum et al., 2020), namely mean and variance for acceleration along each axis, and mean, variance, minimum, maximum, and Shannon entropy of the 2-norm of the accelerometer data. A non-overlapping time window of 0.25s was chosen for computing the features by iterating through different window sizes between 0.25s and 8s and choosing the best-performing window size. The ground truth label for the window was taken from the centre, i.e., the label corresponding to time instant 0.125s from the start of the window. A Gaussian kernel with a bandwidth 0.2 was used to compute entropy.

##### 2.2.4.2 Machine Learning Methods

**FIGURE 3:**
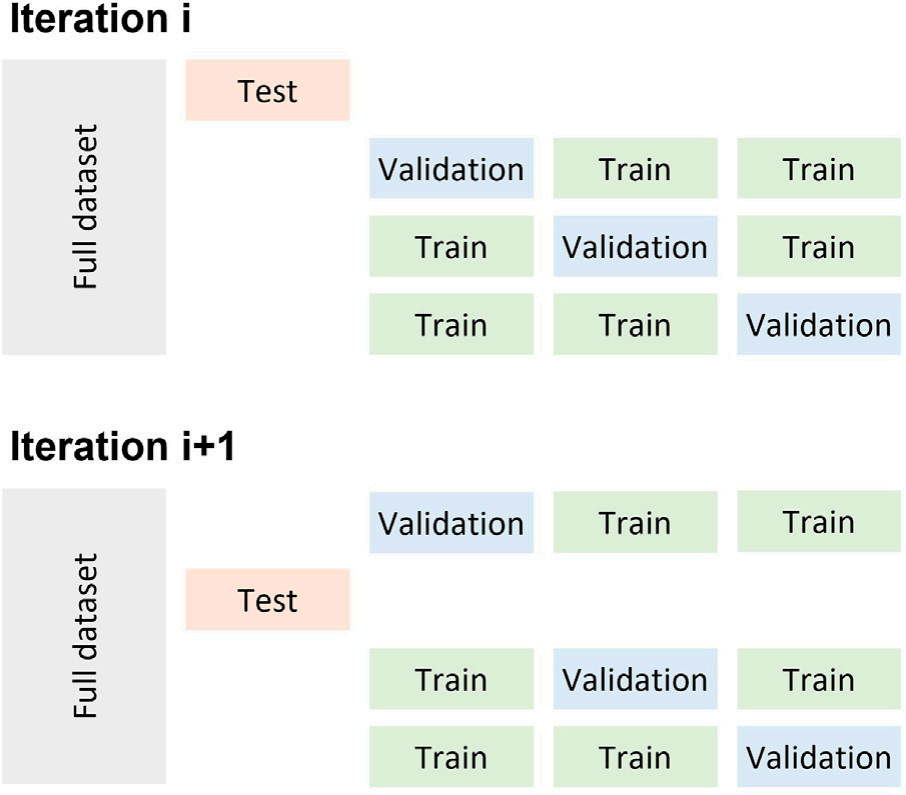
Depiction of the nested cross validation implemented for the different machine learning algorithms.

A Random Forest (RF), a weighted Support Vector Machine (SVM) with a Radial Basis Function kernel, and a three-layer Multi-Layer Perceptron (MLP) were trained on the features and ground truth. The model parameters for the RF and SVM (number of estimators for RF, C and gamma for SVM) were chosen by performing a grid search in a nested cross-validation approach. In nested cross-validation, the model parameters were optimized using every fold in the training set as the validation set (Figure 3). The parameters with the best performance were chosen and tested on the testing set.

*Intra-subject:* Stratified 5-fold cross-validation was used to implement the intra-subject model. To account for the variability in performance due to the different random splits, the intra-subject models were generated by iterating through the train-validate-test process 10 times.

*Inter-subject:* Leave-one-out cross-validation was used to implement the inter-subject model.

All classifiers were implemented using the Scikit-Learn package (Pedregosa et al., 2011).

### 2.3 Interpretation of the Random Forest classifier

To get a handle on the features used by the random forest method, the Gini importance index was computed for the 11 features. The index represents the importance of a feature relative to the other features used in training the model. This analysis was carried out only for the random forest because of the availability of the Gini importance score, and because the random forest performed the best among all methods. To further verify feature importance, three reduced models were trained and validated on the dataset again: 1) mean of $a_{x}$ (1 feature), 2) mean of $a_{x}$ , $a_{y}$ , and $a_{z}$ (3 features), and 3) mean and variance of $a_{x}$ , $a_{y}$ , and $a_{z}$ (6 features).

The Spearman correlation of the mean and variance features with the variables used by TAC and GM methods (e.g., Euler angles of the forearm and activity counts) was also computed. This was done to understand the physical significance of the features.

### 2.4 Effect of data-specific factors on machine learning performance

The nature of the dataset used for developing a machine learning method is a crucial factor in determining performance, arguably as important as choosing a classifier itself. The training dataset used must be a representative sample of the activities and tasks typically performed by a subject in daily life; failing this can drastically affect performance. The training data set must contain sufficient information about the different types of movements of interest to ensure the measure generalizes well to real-world data.

#### 2.4.1 Proportion of functional and non-functional data

The variability in patients’ ability to use their affected limbs resulted in different proportions of functional and non-functional movements in their datasets. To demonstrate the effect of the different proportions, the sensitivities and specificities of the intra-subject random forest models were fit to the percentage of functional data present in the dataset using a linear model, and the slope of this model was tested.

#### 2.4.2 Presence/absence of tasks in testing/training datasets

The current study investigated the effect of presence/absence of selected tasks in training and testing datasets by comparing the performance of the classifier if a certain task is 1) present in both testing and training sets, 2) absent from training set but present in testing set, 3) present in training set but absent from testing set, and 4) absent from both training and testing sets. The tasks chosen for this analysis were opening a bottle, drinking from a cup, and walking (including walking for 25 m, hitting a switch, and opening a door); walking tasks were excluded from the patient data since only two patients had performed them. Additionally, the data segments in between tasks marked as unknown tasks were excluded from this analysis.

##### 2.5 Statistical analysis of measures

The performance of the different UL use measures across different subjects (and different iterations for intra-subject models) are presented as the ‘sensitivity’ vs. ‘1-specificity’ plots. The measures were also compared using the Youden index (Youden, 1950), a measure of the distance between the top-left corner and the position of the model in the plot, which is given by.

The Youden index for the ideal classifier is one and is 0 for a random classifier.

The results from the different analyses were compared using a one-way ANOVA, and t-tests with Bonferroni correction were performed to examine pairwise differences. The following tests were performed:

1) Comparison of the Youden indices of different types of measures was done by grouping them into three categories: traditional (AC, VM, GM and GMAC), inter-subject machine learning, and intra-subject machine learning measures.
2) Comparisons of the Youden indices of reduced and full models using only the random forest inter- and intra-subject models.
3) Comparison of the sensitivities and specificities of all combinations of presence/absence of a task using only the random forest intra-subject models.

The full dataset used in this study and the code for the analysis are available at https://github.com/biorehab/upper-limb-use-assessment.

## 3 Results

Five patients with mild-to-moderate hemiparesis (mean age of 35.4 ± 13.21 years) and ten right-handed unimpaired controls (mean age of 23.3 ± 3.21 years) participated in the study. The demographic details of the patients are listed in Table 3. The inter- and intra-rater agreement for the FAABOS annotations measured by Gwet’s AC1 agreement score were 0.91 ± 0.02 and 0.94 ± 0.02 respectively. The average proportion of data across participants for each label in each group are listed in Table 4.

**TABLE 3:** Demographic details of patients CVA—Cerebrovascular accident; TBI—Traumatic Brain Injury.

Sl. No.	Age (years), sex	Months since injury	Paretic side	Pre-morbid handedness	Cause of injury
1	<30, M	204	Right	Right	TBI
2	40–50, M	3	Left	Right	CVA
3	30–40, M	12	Right	Right	TBI
4	<30, F	7	Left	Right	TBI
5	50–60, M	3	Right	Right	CVA

**TABLE 4:** Average proportion of data corresponding to the different labels

	Arm (%)	Hand (%)	Non-functional (%)
Left	30.24	47.99	21.76
Right	45.89	42.07	12.03
Affected	51.65	9.54	38.81
Unaffected	59.87	16.07	24.07

### 3.1 Which is the best measure for assessing upper limb use?

**FIGURE 4:**
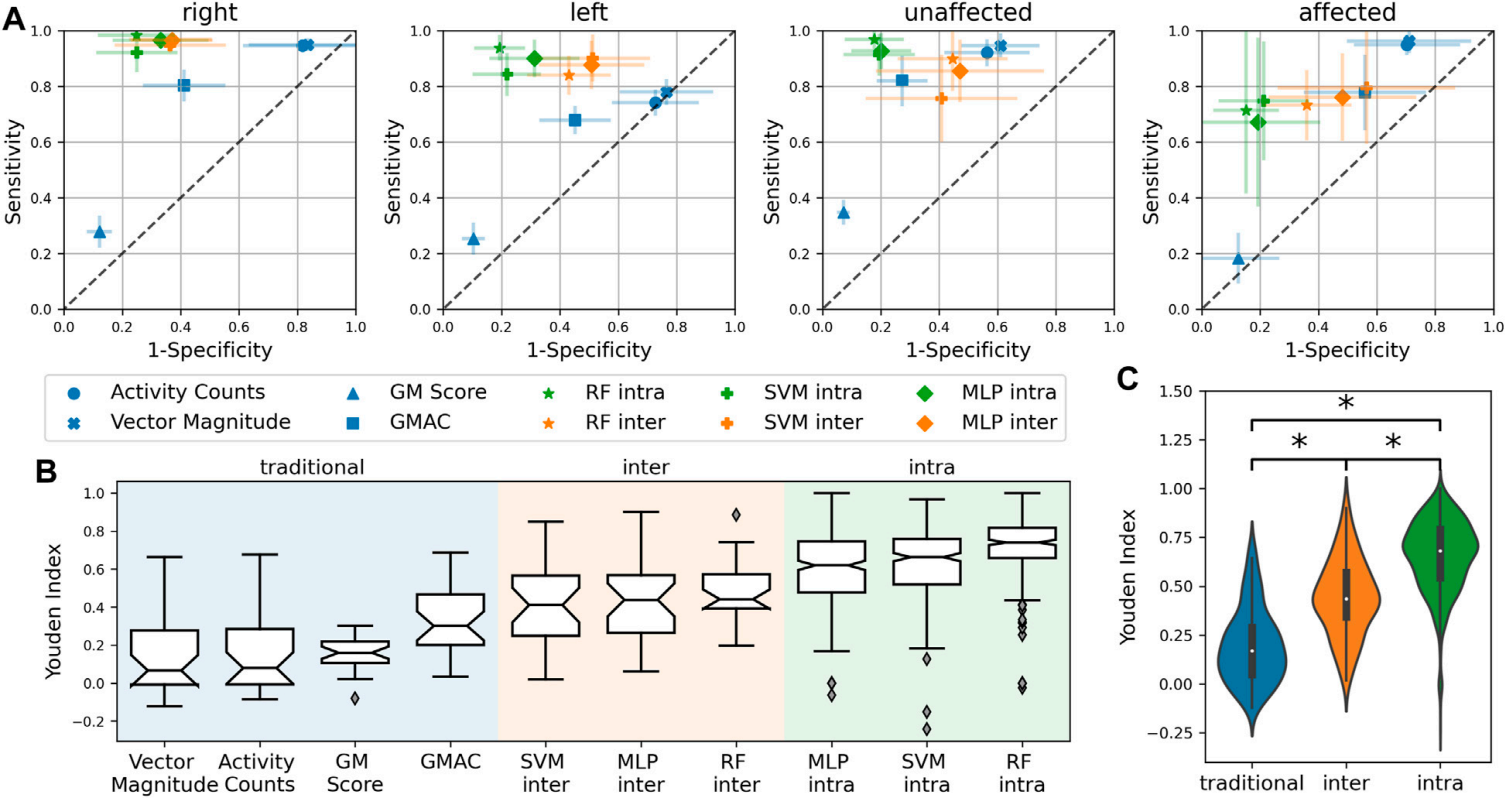
**(A)** Sensitivity vs. ‘1-Specificity’ plots depicting the performance of the different measures. The closer a measure is to the top-left corner, the better its performance. The diagonal dashed gray line depicts the performance of a random classifier. **(B)** Boxplot showing the Youden indices for the measures. **(C)** Statistically significant difference between traditional, inter-subject machine learning and intra-subject machine learning measures. *Significant difference (*p* < 0.05).

The performance of the different measures computed across different subjects (and different iterations for intra-subject models) are presented as the ‘sensitivity’ vs. ‘1-specificity’ plots shown in. Figure 4A, B shows the Youden indices for the different measures. A significant difference was observed between the different types of measures (Figure 4C) (F = 342.5, *p* < 0.0001).

The thresholded activity counting measures had high sensitivity but low specificity with a median Youden index of around 0.07. The GM measure had low sensitivity but high specificity and a median Youden index of 0.16. Thus, confirming that thresholded activity counting measures overestimate, while GM underestimates upper limb use. The amount of activity that the TAC measures pick up is dependent on its threshold. The TAC measures demonstrated in this study have a low threshold of zero that they detect all movements, including non-functional movements, and therefore overestimate use. While the pitch thresholds in the GM algorithm results in a reasonable threshold for functional movements, its threshold for the total change in yaw and pitch may be too restrictive which results in the underestimation of use. GMAC was found to be a reasonable compromise between thresholded activity counting and GM, resulting in a median Youden index of 0.3.

**FIGURE 5:**
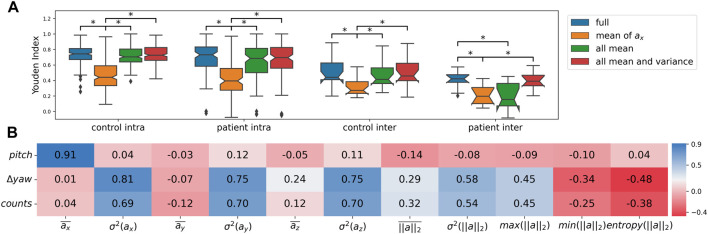
**(A)** Boxplot depicting the Youden indices for the reduced models, *Significant difference (*p* < 0.05); ‘full’ refers to models trained with all features—mean and variance of $a_{x}$ , $a_{y}$ , $a_{z}$ , mean, variance, maximum, minimum and entropy of ${\left\|a\right\|}_{2}$ (11 features), ‘mean of $a_{x}$ ’ are models trained with only the one feature, ‘all mean’ refers to models trained with mean of $a_{x}$ , $a_{y}$ , $a_{z}$ (3 features), ‘all mean and variance’ are models trained with mean and variance of $a_{x}$ , $a_{y}$ , $a_{z}$ (6 features), **(B)** Correlation coefficients between features of the random forest classifier and parameters of GM (Gross Movement score) and TAC (Thresholded Activity Counting).

The machine learning-based upper limb use measures perform better than the traditional measures; intra-subject measures have the best performance (Figure 2C). The RF intra-subject methods had the highest median Youden index of 0.74 among the machine learning measures. Similar to the findings by Lum et al. (2020), the RF method seem to perform slightly better than the SVM; the MLP compared in this study also performs slightly worse than the random forest method. In general, intra-subject models perform better than inter-subject models, which is likely due to inter-subject variability; the inter-subject models for patients are worse than healthy controls (Figure 5A).

Furthermore, we observed that features from the IMU’s gyroscope (e.g., mean and variance of $g_{x}$ , $g_{y}$ , $g_{z}$ ), when added as inputs, did not show statistically significant improvements (*p* > 0.1) in detection performance of the different machine learning algorithms.

#### 3.2 What does the random forest classifier do?

The Gini importance index for the mean and variance of $a_{x}$ , $a_{y}$ , and $a_{z}$ was higher than the other features, with the mean of $a_{x}$ being the most important feature. The Youden indices for the reduced models are depicted in Figure 5A. The intra-subject models and the inter-subject models for controls using just mean of $a_{x}$ was the only model worse than the rest (*p* < 0.05), i.e., using only the mean accelerations achieved similar performance to the full intra-subject model (using all 11 features). However, the acceleration variances were required to achieve similar performance in the inter-subject models for patients; models using just mean of $a_{x}$ and mean of all accelerations showed statistically significant difference when compared to the full model (*p* < 0.05). The SVM and MLP methods showed similar trends.

What do the mean and variance features convey? The Spearman correlation coefficient (shown in Figure 5B) between the forearm pitch angle and mean of $a_{x}$ was 0.91. The coefficient between activity counts and change in forearm yaw angle was around 0.7 with the variance of $a_{x}$ , $a_{y}$ , and $a_{z}$ . Forearm pitch angle indicates forearm’s orientation with respect to ground, while counts and change in yaw indicate the amount of movement in a time window. Therefore, it can be concluded that the random forest method uses a mix of information used by the traditional TAC and GM measures to detect upper limb use. The high performance achieved by only using mean $a_{x}$ suggests the forearm pitch plays a significant role in determining upper limb use, at least in the current dataset.

##### 3.3 How does the nature of the dataset affect a machine learning method’s performance?

There are two data-related aspects that can impact performance. The first is the effect of the proportion of the two classes of movements (functional *versus* non-functional), and the second is the effect of the tasks present in the dataset.

#### 3.3.1 Proportion of functional and non-functional data

**FIGURE 6:**
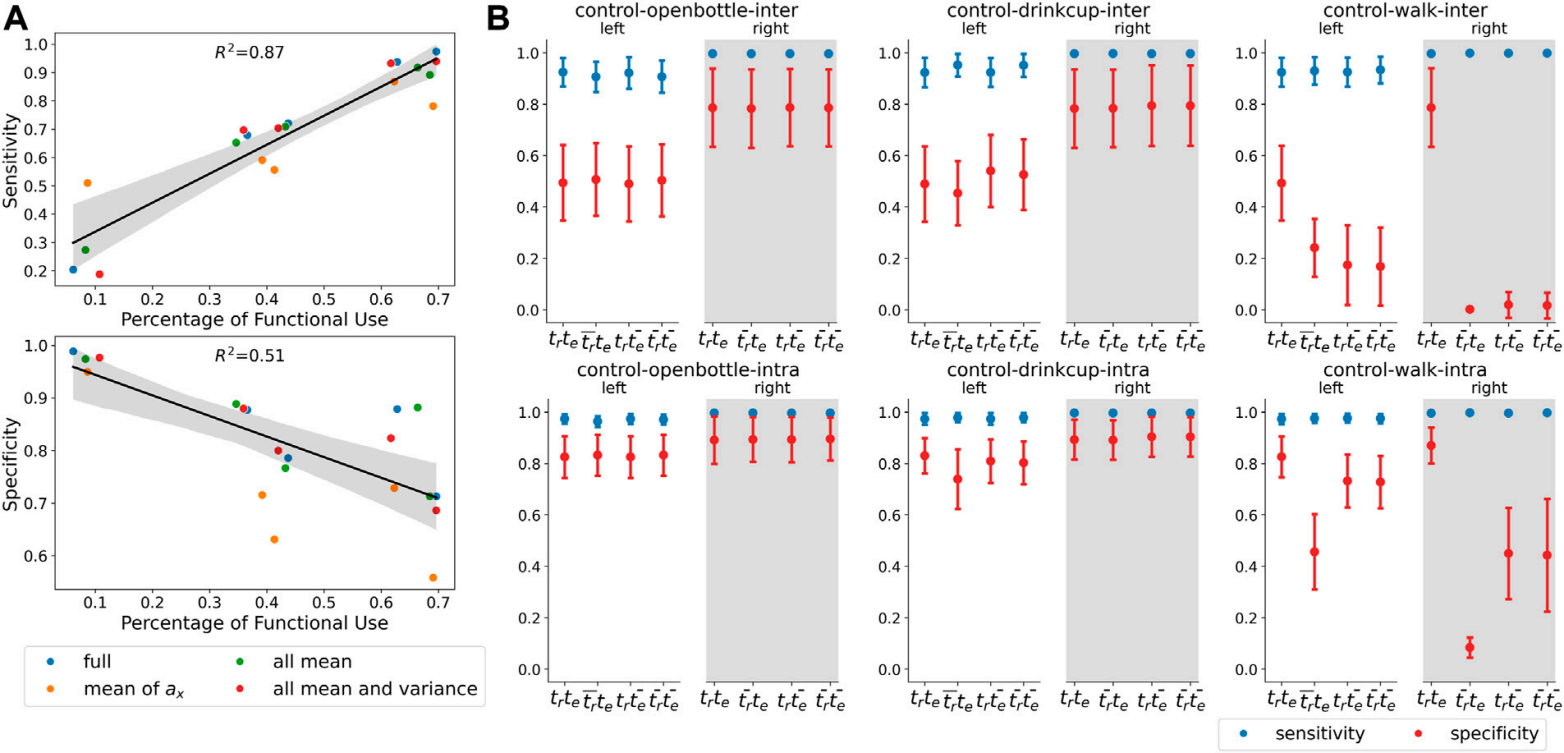
**(A)** Sensitivity and specificity obtained from intra-subject RF classifiers; ‘full’ refers to models trained with all features—mean and variance of $a_{x}$ , $a_{y}$ , $a_{z}$ , mean, variance, maximum, minimum and entropy of ${\left\|a\right\|}_{2}$ (11 features), ‘mean of $a_{x}$ ’ are models trained with only the one feature, ‘all mean’ refers to models trained with mean of $a_{x}$ , $a_{y}$ , $a_{z}$ (3 features), ‘all mean and variance’ are models trained with mean and variance of $a_{x}$ , $a_{y}$ , $a_{z}$ (6 features), **(B)** Sensitivity and specificity obtained when certain tasks were removed from the train and test sets. Changes in sensitivity were found to be statistically insignificant in all tasks except in intra-subject models for opening bottle task for the left hand (F = 6.05, *p* = 0.0005). Task labels ‘openbottle’ corresponds to ‘Open a bottle’, ‘drinkcup’ corresponds to ‘Drinking from a teacup with handles’ and ‘walk’ corresponds to ‘Walk 25 m’, ‘Hit a light switch’ and ‘Open a door’.

The performance variance of the machine learning methods was high for the affected arm of patients due to differences in the amount of functional use in the data for the different patients. The sensitivity and specificity of the intra-subject machine learning models for a patient were proportional to the amount of functional and non-functional movements in the dataset, respectively. This is depicted in Figure 6A for the patient data. Thus, training on a large dataset with an equal amount of functional and non-functional movement data can significantly improve performance.

#### 3.3.2 Presence/absence of tasks in testing/training datasets

Figure 6B shows the sensitivity and specificity of the random forest method (with all 11 features) employing different train and test datasets. The presence of a particular task in the train set is denoted by $t_{r}$ and its presence in the test set is denoted by $t_{e}$ ; their absence is denoted by $\bar{t_{r}}$ and $\bar{t_{e}}$ , respectively. The following observations were made from the results obtained (Figure 6B).

1) There aren't significant changes in the sensitivity under the different conditions for any of the tasks.
2) Specificities for the functional tasks opening a bottle and drinking from a cup do not change much under the different conditions. However, there is a drop in specificity under $\bar{t_{r}}t_{e}$ for drinking from a cup for the intra-subject model for the left-hand data (F = 19.1, p < 0.0001). This could be because almost all control participants used only their right hand for this task, resulting in mostly non-functional data in the left-hand data.
3) There is a significant drop in the specificity for walking tasks under $t_{r}t_{e}$ , $t_{r}t_{e}$ and $t_{r}t_{e}$ , particularly for $t_{r}t_{e}$ when compared to the baseline ( $t_{r}t_{e}$ ) (p < 0.0001).

The results obtained for the patient data at the individual level were varied. Each patient performed different and fewer tasks, and so, meaningful conclusions about the tasks in the dataset could not be made.

## 4 Discussion

The current study compared the sensitivity and specificity of existing measures for quantifying upper limb use—a fundamental construct in assessing upper limb functioning (David et al., 2021b). The results from the comparative analysis show that the machine learning-based measures are better than the other measures presented here and are a promising approach for upper limb use assessment using IMUs. Our analysis, using an independent dataset, confirms the results reported by Lum et al. (Lum et al., 2020): 1) the random forest method is slightly better than SVM in detecting upper limb use; we also found that it is also slightly better than an MLP neural network. 2) Intra-subject machine learning models are better than inter-subject models, which is due to the inter-subject differences in the movement patterns. Another possible reason for the reduced performance of the inter-subject models could be the small number of subjects included for training. However, it is unclear why random forests perform slightly better than the other machine learning methods. Similar observations about random forests are seen in other applications (Biau and Scornet, 2016).

Although the improved performance in upper limb use assessment through machine learning methods is valuable in practice, the black-box nature of these methods obscures their mechanism of operation. Traditional measures (TAC and GM), despite their poor performance, offer an intuitive explanation of their classification since they employ interpretable quantities such as counts, forearm pitch, and yaw. Previous work by Lum et al. employed models using 11 accelerometer features and did not attempt to understand the roles of these different features and their physical significance. Understanding the most relevant and important movement features can guide the optimal selection of sensors for measurements and further improve measure performance. The current study shows that the random forest method employs a combination of arm orientation (mean acceleration features) and the amount of movement (variance of acceleration) to estimate upper limb use. Either GM or TAC does not measure up to the random forest method as they do not use all the relevant information and have relatively simple decision rules. However, an advantage of these traditional methods is that they do not require any training, unlike the machine learning methods. The hybrid GMAC measure performed very closely to the inter-subject machine learning models, indicating that it might still be useful in the absence of training datasets required for employing machine learning methods. Additionally, GMAC can be computed with only accelerometers since it only employs pitch and activity counts. However, the detection ability of the intra-subject models is unmatched owing to the differences between subjects, especially in those with hemiparesis.

Most functional movements were performed on top of a table in the current dataset, while walking formed a significant portion of the non-functional movements class. Therefore, it is no surprise that the pitch of the arm (mean acceleration along *x*-axis) was an important feature in classifying movements. In another dataset where only tabletop tasks are included, the pitch may not play as significant a role, and activity counts could be the determinant in distinguishing between functional movements and rest.

The observations made in this study about the nature of a dataset are preliminary, largely because of the limited number and variety of tasks included in the experiment. The observed results on the impact of the presence/absence of a particular task on a measure’s performance can be explained by two factors: 1) how well represented the movements of this particular task are in that of the other tasks in the dataset, and 2) how much the proportion of the functional class is affected by the presence or absence of a particular task in the dataset. Tasks that have similar movement patterns and similar proportions of functional movements to other tasks can be removed from the dataset without losing performance. But tasks with unique movement patterns and different proportions of functional movements must be included in the training dataset to ensure good performance for a machine learning method. It can be safely concluded that the choice of tasks for the training dataset has an integral part in determining the performance of machine learning-based upper limb use measures. Machine learning methods should ideally be trained on a sample representative of daily life behavior, consisting of similar tasks and proportions of functional and non-functional movements. The variability across subjects in everyday tasks and the amount of functional activity compels the use of intra-subject models for classifying upper limb movements. However, training and deploying these intra-subject models in practice is cumbersome, primarily because of the arduous process of manual ground truth labeling. With a much larger dataset with more participants, it is possible that inter-subject model performance may improve. In which case, pre-trained, generalized models can be deployed without tuning for individual subjects.

This work compared upper limb use measures that employed just two wrist-worn IMU sensors. Continuous and objective assessment of upper limb use can be done in an unobtrusive manner using wrist-worn sensors. The use of additional sensors to filter out walking movements improves performance but is likely to impact usability and adherence (Leuenberger et al., 2017). One of the limitations of detecting use with wrist-worn IMU sensors is that only functional movements can be detected but the differences between functional and non-functional postures cannot be distinguished. For example, resting the forearm on a table will be indistinguishable from holding down a book to keep the pages from turning.

The focus of this work is only on upper limb use, a construct that is useful in evaluating other important usage constructs. Our previous work (David et al., 2021b) expounds on a framework for the assessment of upper limb functioning using sensors and elaborates on other constructs such as intensity of use, functional workspace, movement quality, etc.

The relatively small dataset size used in this current study is its main shortcoming. The dataset contained 10 unimpaired and five stroke survivors with hemiparesis carrying out a set of daily activities; stroke survivors did not perform some of the tasks due to difficulty in performing them. There were also variations in the percentages of functional and non-functional movements within the dataset. Nevertheless, the agreement of the results with that of Lum et al. (2020) restores confidence in the results, despite the small dataset used. Another limitation of the study is that participants were not given food or drink during the eating and drinking activities. The nature of the mimicked movements might have been altered since the risk of spilling food or drink was removed.

The use of machine learning-based measures for quantifying upper limb use seems to be the way forward. Future work in this space must focus on improving the performance of machine learning methods to deploy them in clinical practice efficiently. We propose the following two activities that are worth pursuing:

1) Developing a large, open, annotated dataset with unimpaired and people with impaired movement abilities, performing a wide range of daily activities wearing different types of sensors (at least wrist worn IMUs). This can stimulate work on developing and validating optimal classifiers with high accuracy and reliability. The proposed dataset must have a good sample of the types of movements expected from patients in daily life and a good proportion of functional and non-functional movements of interest.
2) Automatic annotation of functional/non-functional movements from video recordings using an RGBD camera must be explored to eliminate the cumbersome manual labelling process. Recent work has shown that data from multiple IMUs can detect different functional primitives of complex upper limb movements (Parnandi et al., 2021). Thus, it is likely that pose estimates obtained from an RGBD camera can be used for automatically classifying functional and non-functional movements, along with different task types as well.

We believe that exploring these two avenues will help make sensor-based upper limb use detection highly accurate and efficient for routine clinical use.

## 5 Conclusion

This paper presented a detailed comparison of existing measures to quantify upper limb use and confirms previous findings that an intra-subject random forest measure outperforms others. Among the traditional measures, the hybrid GMAC measure, introduced in this study, outperforms all other traditional measures. Thus, the GMAC is an appropriate alternative in the absence of data for training machine learning measures. The current work also sheds light on the random forest measure, demonstrating that it uses a combination of arm orientation and amount of movement to detect upper limb use. The importance of data-related factors, such as class proportion and types of tasks, on the performance of machine learning measures, was also demonstrated. We strongly believe that this study is a step towards understanding and optimizing measures for upper limb use assessment using wearable sensors.

## Data Availability

The datasets presented in this study can be found in online repositories. The names of the repository/repositories and accession number(s) can be found in the article/supplementary material.

## References

[B1] Bailey R. R. Klaesner J. W. Lang C. E. (2014). An accelerometry-based methodology for assessment of real-world bilateral upper extremity activity. PLoS One 9, e103135. 10.1371/journal.pone.0103135 25068258 PMC4113366

[B2] Biau G. Scornet E. (2016). A random forest guided tour. TEST 25 (2), 197–227. 10.1007/S11749-016-0481-7

[B3] Bochniewicz E. M. Emmer G. McLeod A. Barth J. Dromerick A. W. Lum P. (2017). Measuring functional arm movement after stroke using a single wrist-worn sensor and machine learning. J. Stroke Cerebrovasc. Dis. 26, 2880–2887. 10.1016/j.jstrokecerebrovasdis.2017.07.004 28781056

[B4] BrØnd J. C. Andersen L. B. Arvidsson D. (2017). Generating ActiGraph counts from raw acceleration recorded by an alternative monitor. Med. Sci. Sports Exerc. 49 (11), 2351–2360. 10.1249/MSS.0000000000001344 28604558

[B5] David A. ReethaJanetSureka S. Gayathri S. Annamalai S. J. Samuelkamleshkumar S. Kuruvilla A. (2021). Quantification of the relative arm use in patients with hemiparesis using inertial measurement units. J. Rehabil. Assist. Technol. Eng. 8, 20556683211019694. 10.1177/20556683211019694 34290880 PMC8273871

[B6] David A. Subash T. Varadhan S. K. M. Melendez-Calderon A. Balasubramanian S. (2021)., A framework for sensor-based assessment of upper-limb functioning in hemiparesis. Front. Hum. Neurosci., 15. July, 1–22. 10.3389/fnhum.2021.667509 PMC8341809 34366809

[B7] De Lucena D. S. Rowe J. Chan V. Reinkensmeyer D. J. (2021). Magnetically counting hand movements: Validation of a calibration-free algorithm and application to testing the threshold hypothesis of real-world hand use after stroke. Sensors 21 (4), 1502–1519. 10.3390/s21041502 33671505 PMC7926537

[B8] De Lucena D. S. Stoller O. Rowe J. B. Chan V. Reinkensmeyer D. J., “Wearable sensing for rehabilitation after stroke: Bimanual jerk asymmetry encodes unique information about the variability of upper extremity recovery,” 2017, 10.1109/ICORR.2017.8009477 28814049

[B9] Friedman N. Rowe J. B. Reinkensmeyer D. J. Bachman M. (2014). The manumeter: A wearable device for monitoring daily use of the wrist and fingers. IEEE J. Biomed. Health Inf. 18 (6), 1804–1812, Nov. 10.1109/JBHI.2014.2329841 25014974

[B10] Laput G. Harrison C. (2019). Sensing fine-grained hand activity with smartwatches. Conf. Hum. Factors Comput. Syst. - Proc., 1–13. 10.1145/3290605.3300568

[B11] Laput G. Xiao R. Harrison C. (2016). ViBand: High-fidelity bio-acoustic sensing using commodity smartwatch accelerometers. UIST 2016 - Proc. 29th Annu. Symp. User Interface Softw. Technol., 321–333. 10.1145/2984511.2984582

[B12] Leuenberger K. Gonzenbach R. Wachter S. Luft A. Gassert R. (2017). A method to qualitatively assess arm use in stroke survivors in the home environment. Med. Biol. Eng. Comput. 55, 141–150. 10.1007/s11517-016-1496-7 27106757 PMC5222943

[B13] Lum P. S. Shu L. Bochniewicz E. M. Tran T. Chang L. C. Barth J. (2020). Improving accelerometry-based measurement of functional use of the upper extremity after stroke: Machine learning versus counts threshold method. Neurorehabil. Neural Repair 34 (12), 1078–1087. 10.1177/1545968320962483 33150830 PMC7704838

[B14] Madgwick S. O. H. Harrison A. J. L. Vaidyanathan R. (June 2011). Estimation of IMU and MARG orientation using a gradient descent algorithm. Proceedings of the 2011 IEEE International Conference on Rehabilitation Robotics, Zurich, Switzerland, 10.1109/ICORR.2011.5975346 22275550

[B15] Mahony R. Hamel T. Pflimlin J. M. (2008). Nonlinear complementary filters on the special orthogonal group. IEEE Trans. Autom. Contr. 53, 1203–1218. 10.1109/TAC.2008.923738

[B16] Malešević N. Wang C. F. Rich K. Antfolk C. (July 2019). Fall prevention for elderly people using radar sensor. Feasibility Study. Proceedings of the RESNA Annual Conference - 2019, Toronto, Canada,

[B17] McLeod A. Bochniewicz E. M. Lum P. S. Holley R. J. Emmer G. Dromerick A. W. (2016). Using wearable sensors and machine learning models to separate functional upper extremity use from walking-associated arm movements. Arch. Phys. Med. Rehabil. 97, 224–231. 10.1016/j.apmr.2015.08.435 26435302

[B18] Naqvi U. Sherman A. l. (2022). Muscle strength grading. StatPearls. Tampa, FA, USA, https://www.ncbi.nlm.nih.gov/books/NBK436008/. 28613779

[B19] Parnandi A. “PrimSeq: A deep learning-based pipeline to quantitate rehabilitation training,” 2021, http://arxiv.org/abs/2112.11330. 10.1371/journal.pdig.0000044 PMC9681023 36420347

[B20] Pedregosa F., (2011). Scikit-learn: Machine learning in Python. https://dl.acm.org/doi/10.5555/1953048.2078195, 10.5555/1953048

[B21] Regterschot G. R. H. Ribbers G. M. Bussmann J. B. J. (2021). Wearable movement sensors for rehabilitation: From technology to clinical practice. Sensors 21 (14), 4744. 10.3390/S21144744 34300484 PMC8309586

[B22] Shephard R. J. (2003). Limits to the measurement of habitual physical activity by questionnaires. Br. J. Sports Med. 37 (3), 197–206. 10.1136/BJSM.37.3.197 12782543 PMC1724653

[B23] Subash T. David A. Skm V. Balasubramanian S. (2022). “Comparison of wearable sensor based algorithms for upper limb activity detection,” in Converging clinical and engineering research on neurorehabilitation IV, Springer, Heidelberg, Germany, 451–456.

[B24] Totty M. S. Wade E. (2018). Muscle activation and inertial motion data for noninvasive classification of activities of daily living. IEEE Trans. Biomed. Eng. 65 (5), 1069–1076. 10.1109/TBME.2017.2738440 28809669

[B25] Tsai M. F. Wang R. H. Zariffa J. (2021). Identifying hand use and hand roles after stroke using egocentric video. IEEE J. Transl. Eng. Health Med. 9, 2100510. 10.1109/JTEHM.2021.3072347 33889453 PMC8055062

[B26] Uswatte G. Miltner W. H. R. Foo B. Varma M. Moran S. Taub E. (2000). Objective measurement of functional upper-extremity movement using accelerometer recordings transformed with a threshold filter. Stroke 31 (3), 662–667. 10.1161/01.STR.31.3.662 10700501

[B27] Uswatte G. Qadri L. H. (2009). A behavioral observation system for quantifying arm activity in daily life after stroke. Rehabil Psychol, 54, 10.1037/a0017501 PMC2799120 19929121

[B28] Youden W. J. (1950). Index for rating diagnostic tests. Cancer, 3:1. 10.1002/1097-0142 15405679

